# Cisplatin and oleanolic acid Co-loaded pH-sensitive CaCO_3_ nanoparticles for synergistic chemotherapy†

**DOI:** 10.1039/d2ra00742h

**Published:** 2022-05-16

**Authors:** Muhammad Waseem Khan, Chenming Zou, Said Hassan, Fakhar Ud Din, Mahaman Yacoubou Abdoul Razak, Asif Nawaz, Alam Zeb, Abdul Wahab, Sudhair Abbas Bangash

**Affiliations:** Institute of Pharmaceutical Sciences, Khyber Medical University Peshawar Pakistan khanwaseem6065@gmail.com +92-3459146065; School of Pharmacy, Tongji Medical College, Huazhong University of Science and Technology Wuhan Hubei 430030 China; Institute of Biotechnology and Microbiology, Bacha Khan University Charsadda Pakistan; Department of Pharmacy, Quaid-I-Azam University Islamabad 45320 Pakistan; Department of Pathophysiology, School of Basic Medicine, Key Laboratory of Education Ministry of China for Neurological Disorders, Tongji Medical College, Huazhong University of Science and Technology Wuhan 430030 China; Faculty of Pharmacy, Gomal University Dera Ismail Khan Pakistan; Riphah Institute of Pharmaceutical Sciences, Riphah International University Islamabad Pakistan; Department of Pharmacy, Kohat University of Science and Technology Kohat Pakistan; Faculty of Life Science, Department of Pharmacy, Sarhad University of Science and Information Technology Peshawar Pakistan

## Abstract

Despite being one of the most potent anticancer agents, cisplatin (CDDP) clinical usage is limited owing to the acquired resistance and severe adverse effects including nephrotoxicity. The current work has offered a unique approach by designing a pH-sensitive calcium carbonate drug delivery system for CDDP and oleanolic acid (OA) co-delivery, with an enhanced tumor efficacy and reduced unwanted effects. Micro emulsion method was employed to generate calcium carbonate cores (CDDP encapsulated) followed by lipid coating along with the OA loading resulting in the generation of lipid-coated cisplatin/oleanolic acid calcium carbonate nanoparticles (CDDP/OA-LCC NPs). *In vitro* biological assays confirmed the synergistic apoptotic effect of CDDP and OA against HepG2 cells. It was further verified *in vivo* through the tumor-bearing nude mice model where NPs exhibited enhanced satisfactory antitumor efficacy in contrast to free drug solutions. *In vivo* pharmacokinetic study demonstrated that a remarkable long circulation time with a constant therapeutic concentration for both drugs could be achieved *via* this drug delivery system. In addition, the *in vivo* imaging study revealed that DiR-loaded NPs were concentrated more in tumors for a longer period of time as compared to other peritoneal tissues in tumor bearing mice, demonstrating the site specificity of the delivery system. On the other hand, hematoxylin and eosin (H&E) staining of Kunming mice kidney tissue sections revealed that OA greatly reduced CDDP induced nephrotoxicity in the formulation. Overall, these results confirmed that our pH-sensitive dual loaded drug delivery system offers a handy direction for effective and safer combination chemotherapy.

## Abstract 2

Despite being one of the most potent anticancer agents, cisplatin (CDDP) clinical usage is limited owing to the acquired resistance and severe adverse effects including nephrotoxicity.

## Graphical abstract

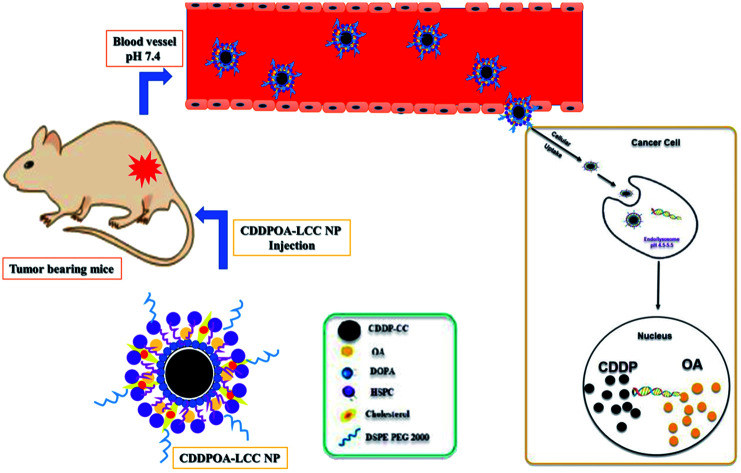

## 1. Introduction

Hepatocellular carcinoma (HCC) is one of the most widespread liver malignant tumors with high morbidity and mortality; with chemotherapy as its main treatment strategy.^1–3^ Cisplatin (*cis*-diamminedichloroplatinum(ii) CDDP), a broad-spectrum antitumor drug, has been extensively used as a model drug for tumor-targeted drug delivery systems. Nevertheless, the acquired resistance and nephrotoxicity of CDDP are still major causes of concern, thereby greatly limiting its chemotherapy applications. Most studies in cell lines and animal models have considered that oxidative stress generated by reactive oxygen species (ROS) overproduction and inflammation mediate the CDDP-induced kidney damage; hence its inhibition may attenuate CDDP-induced nephrotoxicity.^4–7^ CDDP stimulates several antioxidant enzymes transcription *via* nuclear factor erythroid 2-related factor 2 (Nrf2)/antioxidant response element (ARE) signaling pathway deactivation, and induces intracellular injury through chemokines and other pro-inflammatory cytokines release *via* NF-κB activation.^8,9^

A lot of natural compounds with various biological properties have been reported against CDDP driven nephrotoxicity.^10–12^ Among them, oleanolic acid (3β-hydroxyolean-12-en-28-oic acid, OA), a natural pentacyclic triterpenoid with anti-inflammatory and anti-oxidative effects have been reported; basic mechanisms for that includes activation and enhancement of antioxidant and protective enzymes *via* Nrf-2 activation defying the toxic effect of ROS imbalance. OA also deactivates NF-κB pathway minimizing the pro-inflammatory cytokines release associated with CDDP-induced toxicity. In addition, OA and its derivatives have been found to have a potent anti-tumor effect on various cancer cell lines. OA deactivates AKT/mTOR pathway and NF-κB pathway *via* activating AMPK pathway necessary for enhancing cancer cells sensitization and reducing resistance to CDDP chemotherapy.^13–15^

In the recent era, tumor-targeted drug delivery system improvement *via* nanotechnology have presented a remarkable opportunity for improving drug stability and distribution to the targeted tissues with high accuracy and greater retention time, while limiting its toxicity.^16–19^ Combination chemotherapy of co-delivering multiple drugs using nanotechnology is one of such delivery systems which have been employed to maximize therapeutic effects, minimize unwanted effects and develop prognosis through multiple drugs synergistic approach.^20,21^ Owing to OA unique advantages of synergistically enhancing antitumor activity and reducing CDDP-driven nephrotoxicity, we designed a nanoparticulate system for CDDP and OA combination chemotherapy against HCC.

The utilization of stimuli-responsive nano-targeted drug delivery systems especially pH-responsive materials have accelerated tumor therapy development, as the rapid and effective release of drugs at the tumor site can improve the therapeutic effect and reduce the unwanted effects on the healthy tissues. The pH-responsive materials alter their physiochemical characteristics considerably under tumor acidic conditions causing the release of its loaded materials resulting in the selective tumor targeting.^22–24^ Among the pH-responsive materials, calcium carbonate (CC); an inorganic material has exceptional biocompatible and biodegradable properties. CC based nano/micro particles have been utilized as biomaterials for tissue engineering and suitable novel vehicles for an anti-tumor drugs and bioactive proteins. Because of its pH sensitive dissolution, its structure is well-maintained in the neutral settings while slightly acidic atmosphere prompts its payloads release.^25,26^

Owing to this strategy, we have developed cisplatin/oleanolic acid calcium carbonate nanoparticles (CDDP/OA-LCC NPs) having a pH-responsive CC cores, co-delivering CDDP and OA against HCC. Previously, we reported the preparation and characteristics of our optimized CDDP/OA-LCC NPs.^27^ In the present study, we examined our optimized NPs through X-ray diffraction (XRD) analysis and scanning electron microscopy (SEM) to further clarify its physicochemical nature. Fourier transforms infrared (FT-IR) spectroscopy was employed to examine the characteristic functional groups in the developed CC cores. A Kunming mouse model was used to evaluate CDDP and OA *in vivo* pharmacokinetic parameters and OA nephroprotective effect against CDDP induced kidney damage in CDDP/OA-LCC NPs. *In vivo* bio-distribution pattern of the optimized NPs were performed in HepG2 tumor-bearing nude mouse model using Dir-loaded NPs. Finally, the antitumor efficacy was further evaluated *via* the tunnel assay and *in vivo* antitumor treatment in HepG2 tumor-bearing nude mice model.

## 2. Materials and methods

### 2.1. Materials

CDDP (Pt, 65%) and OA of analytical grade were procured. Mono-methoxy polyethylene glycol 2000-distearoyl phosphatidylethanolamine (PEG-DSPE 2000) and 1,2-dioleoyl-in-glycerol-3-phosphate (DOPA) were purchased from Avanti Polar Lipids Inc (Alabaster, AL, USA). Dehydrogenated soya phosphatidylcholine (HSPC) was from Shanghai Advanced Vehicle Technology Ltd (Shanghai, China) while cholesterol from Acros Organics (Geel, Belgium). DMSO, sodium carbonate, sodium chloride, silver Nitrate (AgNO_3_), ethanol, chloroform and methanol (AR grade) were purchased from Sinopharm Chemical Reagent Co, Shanghai, China. Igepal CO-520, PBS, DMEM and cyclohexane from Biosharp, Anhui, China; fetal bovine serum (FBS) were obtained from Zhejiang Tianhang Biological Technology Co., Ltd. (Hangzhou, China). DiR was purchased from AAT Bioquest, Inc. Sunnyvale, CA. All the reagents were used without any further purification unless specified.

### 2.2. Cell lines

HepG2 cells supplied by KeyGen, China were seeded with high glucose DMEM medium, fetal bovine serum, penicillin (100 U mL^−1^) and streptomycin (100 mg mL^−1^) in the incubators (5% CO_2_ and 95% air) at 37 °C.

### 2.3. Animals

Balb/c nude mice (6–8 weeks old, female, average body weight 20.0 g) and Kunming mice (6–8 weeks, female, average body weight 20.0 g) obtained from Beijing Vital River Laboratory Animal Technology Co., Ltd. (Beijing, China) and the Animal Care Facility Centre of Huazhong University of Science and Technology, Wuhan, P. R. China respectively were placed at animal care center with food and water provided *at libitum*. All animal procedures were performed in accordance with the guidelines of “The Institutional Animal Care and Use Committee at Tongji Medical College, Huazhong University of Science and Technology” and approved by the Animal Ethics Committee of “Huazhong University of Science and Technology, Wuhan, China”.

### 2.4. CDDP/OA-LCC NPs preparation

#### 2.4.1 Preparation of CDDP-CC cores

CDDP/OA-LCC NPs were prepared in two steps. In the first step, CDDP-CC cores were prepared followed by the outer lipid coating in the lateral part. The CDDP-CC cores were developed in accordance with the previously reported literature with slight modifications.^28,29^ Two water-in-oil micro emulsions were prepared; (i) calcium emulsion: Briefly, CaCl_2_ aqueous solution (500 mM) was dispersed in oil phase (cyclohexane/Igepal CO-520); (ii) carbonate emulsion: the carbonate part was prepared by dispersing Na_2_CO_3_ (250 mM) aqueous solution in a separate oil phase (cyclohexane/Igepal CO-520). CDDP pro-drug solution prepared in accordance with the previous reported method^29^ and dioleoylphosphatydic acid (DOPA) in chloroform was also added to the carbonate phase. The two oil phases were later mixed together for about 30 minutes. Finally, absolute ethanol was added to break the micro-emulsion system followed by centrifugation (12 000*g* for 30 min) to remove the surfactants, cyclohexane and to collect the pellets. The pellets were finally washed with absolute ethanol (2–3 times) for any DOPA and cyclohexane residual removal, later collected in chloroform and stored in a glass vial.

### 2.4.2 Outer lipid coating

For the outer lipid coating, HSPC : cholesterol : DSPE-PEG-2000 (at a molar ratio of 11 : 1 : 1 mM respectively) were dispersed in chloroform first, followed by its removal *via* rotary evaporator.

Hence, to prepare CDDP/OA-LCC NPs, CDDP-CC cores solution (1 mL), OA solution (695 μL, 4 mg mL^−1^, ethanol) and lipids were dispersed in chloroform. After the chloroform was removed by rotary evaporation, residual lipids were dispersed in PBS (pH 7.4) or H_2_O to generate CDDP/OA-LCC NPs. It should be noted that blank CC cores were used to prepare oleanolic acid-lipid coated calcium carbonate nanoparticles (OA-LCC NPs).

However, for the preparation of a fluorescent DiR-loaded-LCC NPs used during the *in vivo* biodistribution analysis of NPs, same procedure of preparation was followed with the only difference of OA being replaced by DiR dye in the formulation. For the detailed description of the preparation method, please refer to our previously reported literature.^27^

### 2.5. Characterization of NPs

#### 2.5.1. FT-IR spectroscopy

The infrared spectra of the freeze-dried CC NPs were recorded with FTIR spectrophotometer (Bruker Vertex 70; Karlsruhe, Baden-Wurttemberg, Germany) following pelletization with KBr.

#### 2.5.2. XRD analysis

The phase structure of the freeze-dried CC NPs was investigated *via* conducting X-ray diffraction (2 *θ* from 10 to 70°) analysis through PANalytical Xpert PRO diffractometer with Cu-Kα radiation (Shimadzu, Kyoto, Japan, XRD-7000).

#### 2.5.3. Particle size, polydispersity index, and zeta potential measurement

NPs physicochemical characteristics were examined through a Zeta PALS instrument (Brookhaven Instruments, Austin, TX). The NPs formulation was diluted with PBS (pH 7.4) and was sonicated for 5 min before the readings being taken.

#### 2.5.4. Entrapment efficiency and *in vitro* drug release study of NPs

Platinum contents in the NPs were measured through atomic absorption spectroscopy (AAS) SpectrAA-24OFS Atomic Absorption Spectrometer (Varin, USA) while OA loading was determined by high-performance liquid chromatography (HPLC) (Agilent Infinity 1220 LC System., Germany). Dialysis diffusion bag technique was employed at different pH conditions (pH = 5.5 and 7.4) to examine the pH dependent *in vitro* release profiles of our NPs drug delivery system. For the detailed descriptions, please refer to our previously reported literature.^27^

#### 2.5.5. SEM and elemental composition analysis

A Nano SEM 450 field emission scanning electron microscope (FE-SEM) equipped with an energy dispersive X-ray spectroscopy (EDX) was employed to investigate the morphological structure and elemental composition of the developed NPs samples (freeze dried).

### 2.6. *In vivo* pharmacokinetics study

To investigate the drugs *in vivo* fate and pharmacokinetics parameters in our optimized CDDP/OA-LCC NPs, Kunming mice model was used. They were divided into three groups (*n* = 5, average weight 22 ± 2 g) and kept fasted for at least 12 h before the drug administration. Group 1 received CDDP-sol intravenously (iv) *via* tail vein, group 2 received CDDP/OA-LCC NPs iv while group 3 received OA-sol *via* intraperitoneally (ip). Both CDDP and OA were administered at a dose of 7.5 mg kg^−1^ b. wt and 20 mg kg^−1^ b. wt respectively. At each pre-selected reading points (0.5, 1, 2, 4, 6, 8, 12 and 24 h), blood samples were collected from mice by removing eyeballs into the tubes containing heparin and were kept at 20 °C before being analyzed. For determining CDDP concentration, whole blood samples were decomposed on heating in nitric acid and the platinum concentration was calculated by AAS. For determining OA concentration, blood samples were centrifuged (3000*g*, 10 min) to get the plasma and the drug concentration was determined through LC-MS analysis. The chromatographic conditions employed included a chromatographic column (Thermo Hypersil GOLD HILIC 100 × 2.1 mm, 1.9 μm) with a current speed of 0.3 mL min^−1^ and methanol as a washing liquid. Formic acid (0.1%) and acetonitrile formate (0.1%) were used as an aqueous and organic phase respectively. The column temperature was maintained at 30 °C while that of automatic sampler at 8 °C. Sample volume of automatic sampler was 5.00 microL, injection needle height was 2.00 mm while the immersion time of automatic injector during needle insertion cleaning was 3.00 ms.

Finally, the pharmacokinetic parameters were calculated through a non-compartment analysis by Pharsight WinNonlin analysis software.

### 2.7. *In vivo* imaging and biodistribution analysis

DiR, a near-infrared fluorescent and a member of indocarbocyanine dye family frequently used for the *in vivo* tracking^30^ was used for *in vivo* imaging and biodistribution analysis of our drug delivery vehicle *i.e.* calcium carbonate nanoparticles (CC NPs). Female Balb/c nude mice bearing subcutaneous HepG2 tumors were used for this investigation. About 200 μL of DiR-loaded CC NPs (explained in Section 2.4.2; Preparation of CDDP/OA-LCC NPs) were injected through the tail vein to each mouse with the same dosage of DiR (50 μg kg^−1^). The IVIS Lumina XR system equipped with a 150 W quartz halogen lamp and a 1 mW power scanning laser (Caliper Life Sciences, Hopkinton, USA) was employed to obtain fluorescent images due to DiR signals at predetermined time intervals (6, 12 and 24 h). Mice were anesthetized by 10% hydrated chloral solution before and during the imaging process. After the living imaging of mice, they were sacrificed and their major organs (hearts, livers, spleens, lungs, kidneys and tumors) were collected and imaged to measure fluorescence signal intensities. All the results were analyzed by IVIS software.

### 2.8. OA Nephroprotective effect against CDDP induced injury in mice

#### 2.8.1 Experimental design

To examine the OA protective nature on CDDP-induced kidney injury in mice, seven groups of mice were made (*n* = 5). Group 1: an untreated control given normal saline iv; Group 2: given corn oil orally for 7 days (5 mL kg^−1^ body weight (b. wt)); Group 3: received OA-sol injection ip for 7 days and a single CDDP-sol injection iv 1 hour after the OA-sol injection (on 2^nd^ day). Group 4 received the same doses of drugs of group 3 but in NPs dosage form. Group 5 and Group 6 received a single CDDP-sol and CDDP-LCC NPs injection iv respectively on the 2nd day of treatment. Whereas, the 7th group received a single CDDP/OA-LCC NPs injection iv on the 2nd day of the study period. Both CDDP and OA were administered at a dose of 7.5 mg kg^−1^ b. wt and 20 mg kg^−1^ b. wt respectively.

Finally, the mice were euthanized and their kidney tissue samples were collected for toxicity evaluation *via* H&E staining. During the staining, samples were initially fixed in paraformaldehyde (4%) for 3 h, followed by an extensive PBS washing for an overnight. Later they were paraffin embedded and cut into 4 μM thick sections. Finally, the tissue sections were deparaffinised and dehydrated in xylene and ethanol (Sinopharm, Shanghai, China). At the end, they were stained with H&E staining kit (Servicebio) as per instructions to examine any histological alterations through Olympus 1X71 microscope, Japan.

### 2.9. *In vivo* anti-tumor efficacy

The anti-tumor efficacies of individual drug's solutions, their individual NPs and our final NPs were assessed by the xenograft HepG2 human hepatocellular carcinoma on Balb/c nude mice which was established *via* injecting 200 μL HepG2 cells (2 × 10^5^) subcutaneously into the right flank of each mouse. When the tumor volume reached about 80–100 mm^3^, the mice were weighed and randomly divided into 6 groups (*n* = 5). Mice in each group (1–6) were injected with 0.2 mL of saline, CDDP-sol, CDDP-LCC NPs, OA-Sol, OA-LCC NPs and CDDP/OA-LCC NPs respectively, with both CDDP and OA administered at a dose of 0.5 mg kg^−1^ iv and 5 mg kg^−1^ ip respectively. The treatments were carried out every second day for a total of five injections. The tumor length (*L*) and width (*W*) measured *via* a caliper were used to determine the tumor volume through the following equation;

1 Tumor volume = (*L* × *W*^2^)/2 ^31^

whereas, the mice body weights were measured to assess systemic toxicity of the formulations. After the final injection, mice were sacrificed by CO_2_ asphyxiation for tumors excision. A portion of tumor was fixed in 4% formalin for TUNEL assay. Data analysis was performed through GraphPad Prism 5 (GraphPad Software Inc., San Diego, CA) *via* one-way ANOVA followed by *t*-test.

### 2.10. Tunnel assay

Tumor slides after deparaffinization with xylene and a graded alcohol were prefixed with 4% paraformaldehyde. To determine apoptosis in the tumor tissue, the *in situ* terminal deoxynucleotidyl-transferase dTUP nick end labelling (TUNEL) assay were conducted following the manufacturer's instructions (Roche). TUNEL-positive nuclei images were acquired through fluorescence microscope (Olympus 1X71 microscope, Japan).

### 2.11. Statistical analysis

The experimental data are expressed as mean ± standard deviation (SD) and plotted using Origin or GraphPad Prism software. Statistical analysis of the experimental data was performed with one-way ANOVA followed by the *t*-test to compare differences. *P* < 0.05 was considered statistically significant.

## 3. Results and discussion

### 3.1. Preparation of NPs

An overall graphical preparation method for NPs (CDDP/OA-LCC NPs) is demonstrated in ESI Fig. S1.† A micro-emulsion method comprising a nanoprecipitation step was performed to create CDDP-CC cores. The cores pre-coating agent (DOPA) tightly reacts with platinum cations (at the carbonate and calcium ion interface)^32^ resulting in the production of stable precipitates, keeping the nano-precipitates sizes in-check and averts the potential aggregation during the centrifugation. The outer covering lipids were cautiously picked as they greatly influence NPs pharmacokinetics and tissue distribution,^33^ hence HSPC, CHOL and DSPE-PEG-2000 (11 : 1 : 1) coating layer were encrusted onto the CDDP-CC cores for a stable NPs generation; with OA also being added onto our final formulation at this stage. Finally, centrifugation was performed to get rid of the unbound drug and free liposomes from the hydrated CDDP/OA-LCC NPs.

### 3.2. NPs characterization

DLS measurement confirmed the average NPs diameter of 217 ± 20 nm while zeta potential and polydispersity index was found to be −23.7 ± 2 and 0.187 respectively, demonstrating the NPs narrow and homogeneous dispersion. Drug loading was found to be 76 ± 5% and 50 ± 7% (w) for CDDP and OA respectively.

**Fig. 1:**
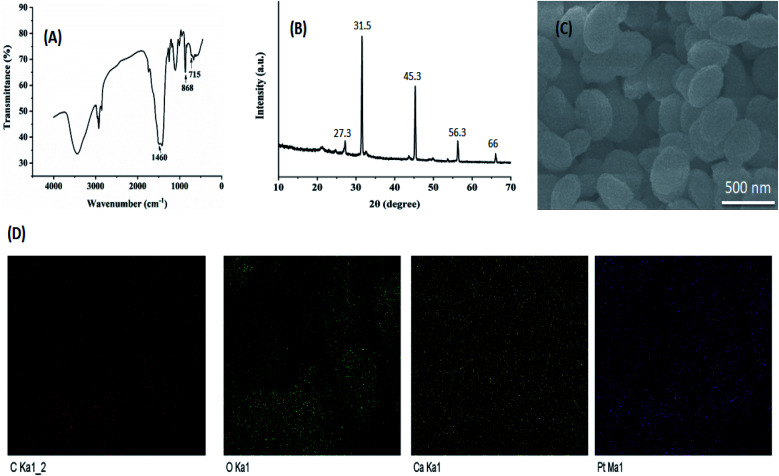
Characterization of NPs. (A) FT-IR spectra of CC NPs. (B) XRD pattern of our optimized formulation (CDDP/OA-LCC NPs). (C) SEM micrographs/images of our optimized formulation (CDDP/OA-LCC NPs), scale bar: 500 nm. (D) The elemental mapping analysis for our optimized formulation (CDDP/OA-LCC NPs).

The FTIR spectra of freeze-dried CC NPs were shown in Fig. 1A. The spectrum showed a characteristic peak at 1460 cm^−1^ due to symmetrical −COO^−^ stretching of carbonate ions and peaks at 868 cm^−1^ and 715 cm^−1^ corresponding to the *ν*_2_ and *ν*_3_ CO_3_^2−^ absorption bands of calcite.^34^ In addition, the crystal phase of the developed NPs was investigated by X-ray diffraction (XRD) analysis. A greater number of broad peaks appeared at 2*θ* (from 10 to 70°) in the XRD pattern (Fig. 1B), indicating the crystalline nature of our optimized NPs.

The surface morphology of the optimized NPs was examined using FESEM as shown in Fig. 1C. FESEM micrographs demonstrated that NPs are generally spherical with overall smooth surfaces and with size ranges in alignment with DLS results. To take further insight into our optimized NPs structure, a high efficiency EDX detector equipped with FESEM was employed to reveal the elemental mapping of CDDP/OA-LCC NPs. EDX micrographs (Fig. 1D) of particles revealed the availability of all the elements in our final NPs.

It was noted that platinum release from NPs at acidic range (pH = 5.5) was noticeably higher than its release at neutral range (pH 7.4) after 72 h, with a cumulative release of 70 ± 4.6% and 28 ± 4.1% respectively as shown in ESI Fig. 2A & B.† On the other hand, no major difference was found in the OA release profile in both neutral and acidic media shown in ESI Fig. 2A and B.†

The alteration in the platinum release profiles might be because due to its encapsulation inside the CC cores which remained stable at neutral pH with a limited release. While at low pH ranges, CC cores collapsed rapidly resulting in the fast and easy release of encapsulated drugs,^35^ which was evident from our data. This pH dependence drug delivery system provided prospects for tumor-targeted delivery as the pH level drops to 5 when the endocytotic vesicles delivered from the cytoplasm (pH 7.4),^36^ hence, making our NPs system an ideal drug carrier to the cancer cells to accomplish high selectivity.

### 3.3. *In vivo* pharmacokinetics study

**Fig. 2:**
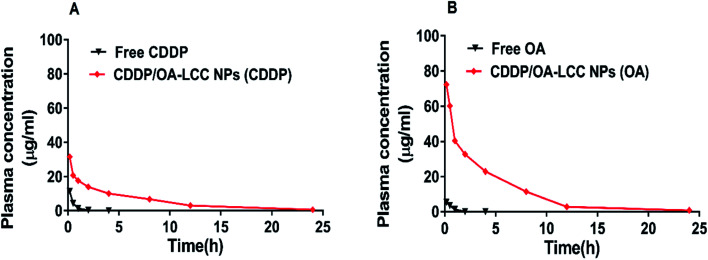
Drug plasma concentration–time profiles. (A) CDDP (B) OA; after CDDP-sol, OA-sol and CDDP/OA-LCC NPs administration to Kunming mice at a dose of 0.5 mg kg^−1^ for CDDP and 5 mg kg^−1^ for OA based on the animal's body weight.

Pharmacokinetic studies greatly help in understanding the mechanism of interactions between anticancer drugs which would affect therapeutic activities or toxic side effects.^37^ For the pharmacokinetics studies, Kunming mice (3 groups; *n* = 5) were used as animal model. CDDP-sol and CDDP/OA-LCC NPs were injected *iv via* the tail vein while OA-sol was administered ip. Drug concentrations in the blood plasma were determined for both CDDP and OA *via* AAS and LC/MS method respectively, with their plasma concentration *versus* time profiles are shown in Fig. 2A and B.

It was noted that CDDP/OA-LCC NPs development considerably altered the pharmacokinetic parameters of both CDDP and OA. In case of free CDDP-sol and free OA-sol injections, the plasma drug concentrations were decreased rapidly and were eliminated rapidly from the body with no drug concentrations observed after about 5–6 h for both free CDDP and OA. On the other hand, our optimized NPs enhanced the circulation time for both CDDP and OA considerably with their plasma concentration being detected at the 24 h terminal point. A longer elimination half-life (*t*_1/2_) was probably due to its ability to avoid elimination from the plasma. The longer plasma mean residence time (MRT) and half-life of both the drugs in our optimized formulation may be of potential benefit since both CDDP and OA have a time-dependent cell cycles and apoptosis mechanisms, hence the extended exposure afforded by longer plasma *t*_1/2_ could result in an increased tissue or tumor concentration. It should also be noted that overall CDDP release was slower than the OA release, demonstrating the pH sensitivity of the CC carrier in our optimized formulation (CDDP/OA-LCC NPs), as it could greatly protect the drug release in the basic medium but release the encapsulated CDDP in the acidic tumour medium greatly enhancing its selectivity and efficacy.

Pharmacokinetic parameters calculated through Pharsight WinNonlin are shown in Table 1. Compared with CDDP-sol group, the *C*_max_ and MRT for CDDP in CDDP/OA-LCC NPs group was increased by about 2.76 and 11.16-folds respectively while for OA in CDDP/OA-LCC NPs group it was increased by 13.15 and 6.31-folds respectively as compared to OA-sol group. In case of other parameters such as AUC and *t*_1/2_, it was also significantly increased for both CDDP and OA in CDDP/OA-LCC NPs group as compare to CDDP- sol and OA-sol groups.

**Table 1:** CDDP and OA pharmacokinetic parameters after free CDDP (0.5 mg kg^−1^, i.v), free OA (5 mg kg^−1^, i.p) and CDDP/OA-LCC NPs i.v (0.5 mg kg; 5 mg kg^−1^) administrations in Kunming mice (*n* = 3)

Formulation	*C*_max_ (μg L^−1^)	MRT_0-∞_ (h)	AUC_0-∞_ (μg h L^−1^)	*t*_1/2_ (h)	CL (mL h^−1^)
Free CDDP	11.45 ± 0.65	0.504 ± 0.046	8.04 ± 0.34	0.74 ± 0.09	1.227 ± 0.053
Free OA	5.50 ± 1.94	0.69 ± 0.03	4.27 ± 0.62	0.66 ± 0.05	23.16 ± 3.14
CDDP/OA-LCC NPs (CDDP)	31.61 ± 2.22	5.627 ± 0.157	140.28 ± 6.70	4.90 ± 0.68	0.069 ± 0.003
CDDP/OA-LCC NPs (OA)	72.36 ± 1.94	4.360 ± 0.327	272.59 ± 8.78	3.83 ± 0.37	0.0361 ± 0.020

Overall, pharmacokinetics data suggests that our optimized NPs formulation could significantly enhance the circulation time and maintain a constant therapeutic concentration for both drugs (CDDP and OA) within the safety ranges throughout the study time period, making it an ideal drug delivery system for synergistic combination chemotherapy.

### 3.4. *In vivo* imaging and bio-distribution analysis

**Fig. 3:**
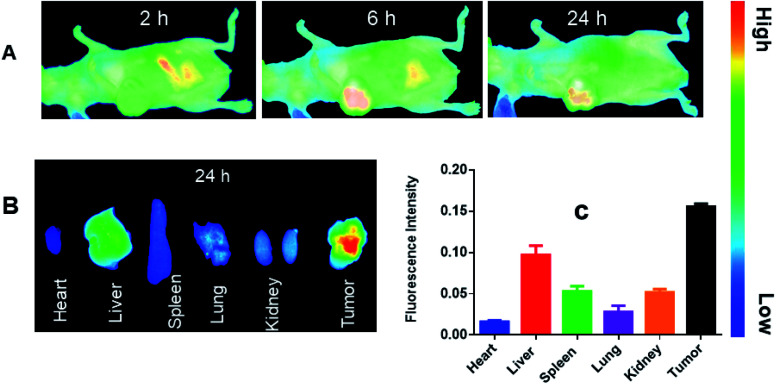
*In vivo* distribution of DiR-loaded CC NPs. (A) *In vivo* whole-body imaging of HepG2 tumor bearing mice after DiR-loaded CC NPs administration at 2 h, 6 h & 24 h postinjection. (B) *Ex vivo* imaging of Dir-loaded CC NPs in major organs and tumor tissues at 24 hours. (C) The distribution of fluorescence intensity in different organs and tumor at 24 h postinjection. Data presented as mean ± SD, *n* = 5.

The *in vivo* bio-distribution and tumor targeting effectiveness of the prepared drug delivery vehicle (LCC-NPs) was evaluated in a xenograft mouse model of human hepatic carcinoma cell lines (HepG2), employing a fluorescent dye, DiR. It is an indocarbocyanine dye; a suitable choice for the *in vivo* bio-distribution studies with the emission wavelength of above 700 nm which greatly eliminates emission interference from the cell background.^38^ NPs containing DiR were *iv* injected into the tumor bearing mice, later imaged at specific time points (2 h, 6 h & 24 h) using IVIS Lumina XR system. From the results shown in Fig. 3A, it was revealed that DiR-loaded LCC-NPs tended to be enriched in the tumor site over a considerable time period, with a maximum intensity at 6 h time point which remained detectable till 24 h post-injection. The increased accumulation of DiR-loaded NPs at the tumor sites demonstrated that our delivery system has the passive tumor-targeting capabilities which might be due enhanced permeation and retention (EPR) effect.^39^ More importantly, the enhanced accumulations of Dir-loaded NPs at the tumors might facilitate synergistic chemotherapy to overcome the resistance of cancer cells.

In addition, ex vivo fluorescent images of excised tissues (Fig. 3B and C) showed a statistically significant higher accumulation of DiR-loaded NPs (24 h post injection) in tumor tissues while a slightly weak fluorescence signals were observed in other peritoneal tissues. The data revealed a reduced systemic exposure of healthy tissues to CDDP making it less toxic to other tissues, as lower accumulation of fluorescence signals was observed in kidney propagating our combination chemotherapy nephroprotective nature and site specificity, making our developed nano-particulate system an ideal candidate for a combination therapy against HCC.

### 3.5. OA alleviates CDDP-induced nephrotoxicity in mice

**Fig. 4:**
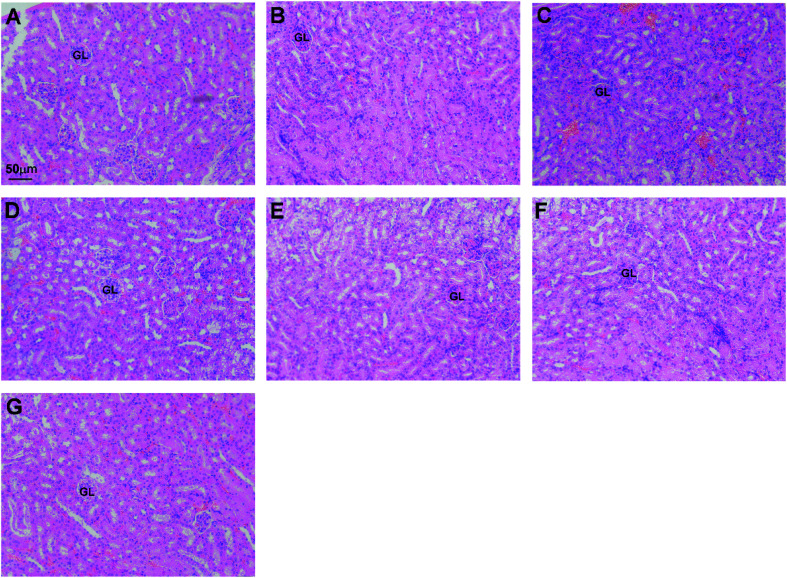
OA alleviates CDDP induced nephrotoxicity. H & E stained kidney tissues scale bars: 50 μm (A) control (NS): normal histological structure of the kidney showing glomerulus with well delineated Bowman's capsule, well organized tubular architecture having normal epithelium and brush borders; (B) corn oil group: having normal histological kidney architecture with mild congestion or increased perfusion; (C and D) OA-sol + CDDP-sol and OA-LCC NPs + CDDP-LCC NPs groups respectively: moderate congestion with little loss of normal nuclei structure; (E and F) CDDP-sol and CDDP-LCC-NP groups respectively: presence of cuboidal epithelium with no prominent or pyknotic nuclei and loss of brush borders, loss of epithelium with tubular necrosis; (G) CDDP/OA-LCC-NPs: relatively less congestion and less toxicity with relatively normal kidney histology.

We next investigated the OA ability to protect CDDP-induced kidney injury from histopathologic changes in the kidney sections after the drugs exposure (Fig. 4). Kidney sections of the control group showed the normal histological structure having a well-defined glomerulus with well delineated Bowman's capsule, well organized tubular architecture with normal epithelium and brush borders (Fig. 4A), while the vehicle (corn oil) group demonstrated a normal histological architecture with mild congestion or increased perfusion (Fig. 4B). On the other hand, CDDP-sol and CDDP-LCC-NP treated groups (Fig. 4E and F) demonstrated the most toxicity with no prominent or pyknotic nuclei and the loss of brush border on cuboidal epithelium, and GL was not well defined and loss of epithelium was observed with tubular necrosis. The mice groups (Fig. 4C and D) showed a moderate congestion with little loss of normal nuclei structure because of the repeated doses of OA-sol and OA-LCC NPs injections before and after CDDP injections (2^nd^ day of treatment) indicating the greater potential nephroprotective effect of OA against CDDP. The mice receiving a single CDDP/OA-LCC NPs injection (Fig. 4G) developed a slender morphological change, though were in a much better shape than CDDP alone treated groups (Fig. 4E and F) indicating the greater safety profile of our optimized formulation for the combination therapy against HCC.

CDDP is used as a chemotherapeutic agent alone or in combination therapy, however its induced nephrotoxicity hinder its broader usage. Both the experimental and clinical data have shown that oxidative stress generated by ROS overproduction plays a pivotal role in CDDP-induced renal damage. ROS acts directly on cell components such as lipids, proteins and DNA, destroying their structure. CDDP has also been reported to deactivate the Nrf2/ARE signaling pathway which stimulates the transcription of several antioxidant enzymes; Phase II detoxifying proteins and phase III efflux transporters which quickly neutralize, detoxify and remove the oxidizing xenobiotics. A lot of evidences have suggested that inflammation too has a vital role in the pathogenesis of CDDP-induced nephrotoxicity.^11,40–43^

It has been reported that CDDP causes intracellular injury prompting the release of damage associated molecular pattern molecules (DAMPS) which act on Toll-like receptors (TLRs). Thereafter, TLRs causes the release of chemokines and other pro-inflammatory cytokines *i.e.* TNF-α and IL-1β *via* multiple pathways such as NF-κB. It has also been reported that NF-κB activation further stimulates the transcription of certain genes such as cyclooxygenase-2 and inducible nitric oxide synthase which sequentially results in inflammation.^44–46^

OA activates and enhances Nrf2 signaling pathway which plays a crucial role in anti-oxidant enzymes regulation and has been reported as a target site for the treatment of CDDP-induced nephrotoxicity. Antioxidant and protective enzymes stimulated by OA through Nrf-2 activation includes glutathione reductase, superoxide dismutase and heme oxygenase-1 defying the toxic effects of ROS imbalance.^47,48^ OA has also been reported for enhancing the amount of hepatic glutathione (GSH) level and GSH: glutathione disulfide ratio (a cellular redox balance indicator). GSH scavenges ROS efficiently, detoxifies xenobiotics and their metabolites.^49^

In our optimized formulation (CDDP/OA-LCC NPs), OA neutralized CDDP induced kidney injury by diminishing its causing factors and played its nephroprotective role. OA significantly attenuated CDDP-induced NF-κB activation reported previously by our group,^27^ leading to the regulation of inflammatory cytokines release and ultimately the nephroprotection.^50,51^

Hence, we can say that OA addition to the CDDP combination chemotherapy in our system (CDDP/OA-LCC NPs) makes it an ideal therapy for combating nephrotoxicity in patients receiving CDDP chemotherapy.

### 3.6. *In vivo* antitumor efficacy and TUNEL assay

**Fig. 5:**
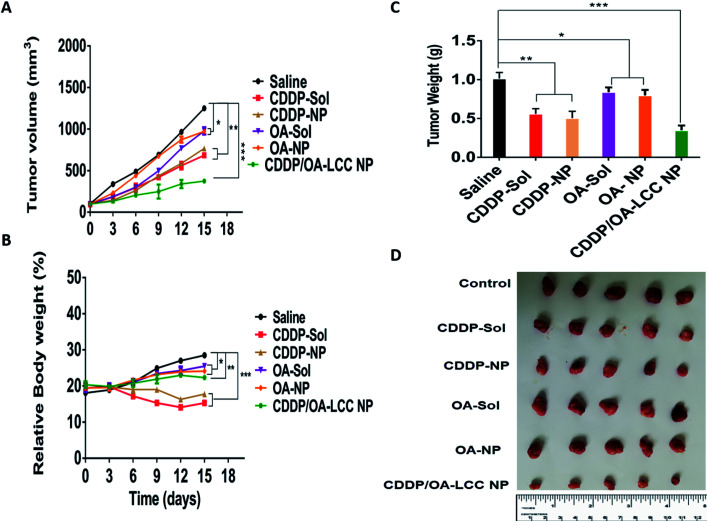
*In vivo* anti-tumor effect on subcutaneous HepG2 xenograft-bearing mice. (A) Tumor growth curves of mice bearing subcutaneous HepG2 tumors treated with different formulations. (B) Body weight variations of mice bearing subcutaneous HepG2 tumors treated with different formulations. (C) Weights of tumors of mice bearing subcutaneous HepG2 tumors treated with different formulations at the end of experiment. Data presented as mean ± SD, *n* = 5. Statistically significant differences between saline group and other groups were marked as *p < 0.05, **p < 0.01, ***p < 0.001. (D) Photographs of the excised tumors of mice bearing subcutaneous HepG2 tumors at the end of the treatment with different formulations.

**Fig. 6:**
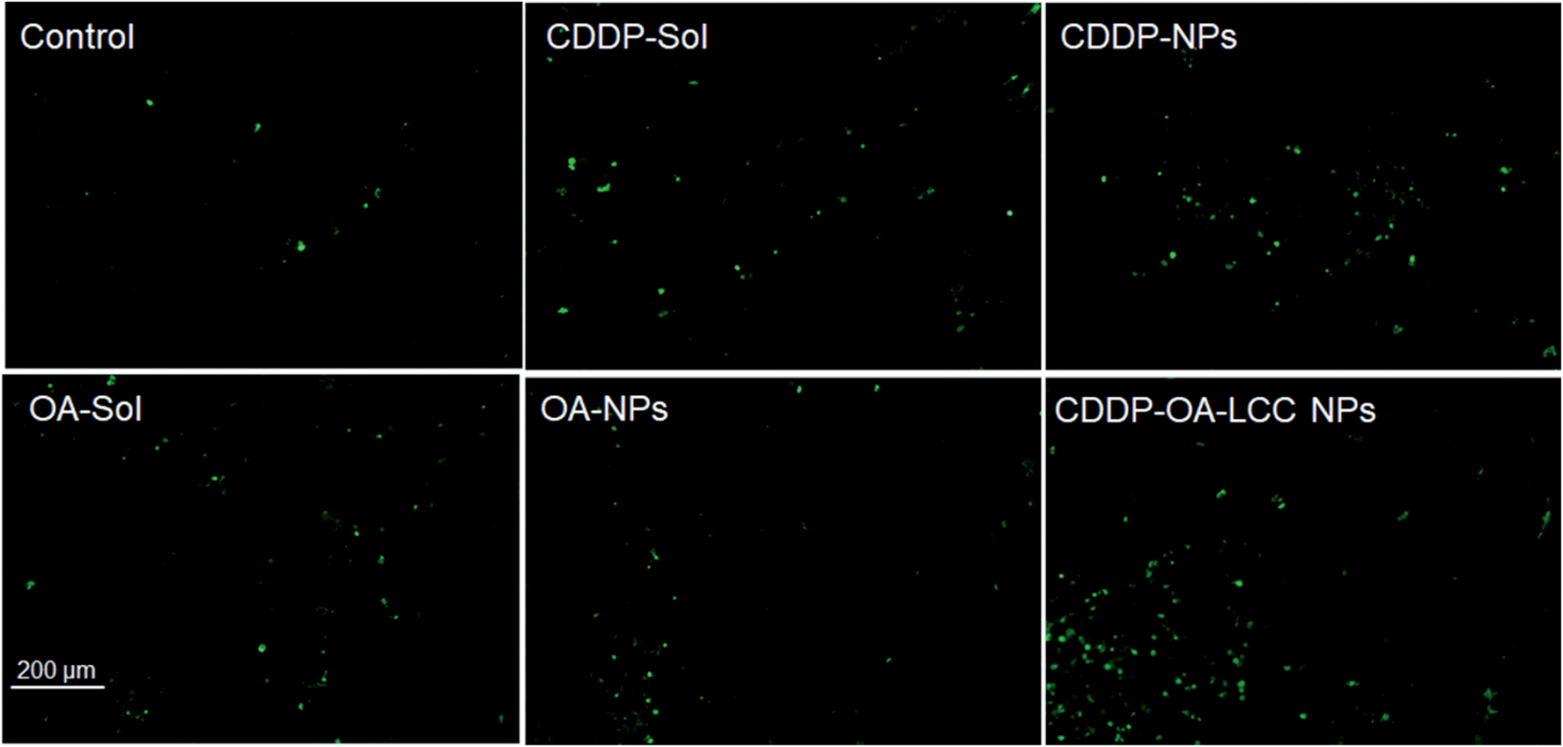
TUNEL assay on HepG2 tumor cells after treatment with different formulations *in vivo*, scale bar: 200 μM.

We used Balb/C nude mice bearing subcutaneous HepG2 tumors to assess the antitumor efficacy of various formulations *i.e.*, normal saline, CDDP-sol and NPs, OA-sol and NPs, and CDDP/OA-LCC NPs, with the results shown in Fig. 5. Mice were regularly checked (every three-day) for the tumor volume and body weights during the treatment course. The tumor volume measured for each group was plotted as a function of time (Fig. 5A). For the normal saline group, tumors sizes gradually increased as a function of time. CDDP (sol, NPs) and OA (sol, NPs) groups demonstrated the tumor growth suppression effect compared to the saline treated groups, with the nanoparticle's groups showed more suppression effect than their corresponding solution groups in accordance with the *in vitro* cell culture results previously reported before by our group,^27^ shown in ESI Fig. S3 & S4,† attributing to the improved retention time and effective internalization of our nanocarriers by the cancer cells. In contrast, our combination therapy treated groups (CDDP/OA-LCC NPs) demonstrated the highest antitumor activity depicting the greater synergistic effect of CDDP and OA in our optimized formulation. In accordance with the *in vitro* cell culture results as reported before by our group,^27^ shown in ESI Fig. S4† where the combined nanoparticles group (CDDP/OA-LCC NPs) have inhibited the cancer cell growth more than any of the testing group depicting the synergistic apoptotic effect of CDDP and OA in our optimized nanoparticulate system. Body weight was the next parameter to examine the toxicity or adverse effects of the formulation As shown in Fig. 5B, CDDP group showed a significant body weight loss as compare to OA treated group or saline group, demonstrating the greater risk associated with CDDP when used alone. However, in our optimized formulation group (CDDP/OA-LCC NPs), the overall CDDP toxicity was greatly minimized with the OA addition, proving the potential safety of the developed NPs. Tumor cell apoptosis was further examined *via* a TUNEL assay after treatment shown in Fig. 6. CDDP-solution and CDDP NPs treated cells showed a comparably greater proportion of DNA fragmentation in contrast to normal saline, OA-solution and OA-NPs treated groups. Whereas, CDDP/OA-LCC NPs treated group showed the highest proportion of DNA fragmentation as compare to the other treated groups. These finding were correlated with the tumor inhibition data shown in Fig. 5 and *in vitro* cell culture data previously published by our group,^27^ shown in ESI Fig. S3 & S4.† The results suggested that OA acted as a capable anticancer agent and played a critical role in the overall synergy with CDDP against HepG2 cells in our optimized NPs.

The basic mechanisms underlying the synergistic effect of CDDP and OA in our combination therapy against HCC is that OA significantly enhances HepG2 cells sensitivity towards CDDP and ultimately enhanced the apoptosis, reported before by our group shown in ESI Fig. S5.†^27^

One of the possible mechanism underlining this is P13K/Akt/mTOR pathway; a major signal transduction cascade hyper-activated in most cancers whose activation leads to cancer cells survival and its proliferation *via* attenuating the antitumor effect of chemotherapeutics,^52,53^ performs a crucial part in cancer cells CDDP resistance and ultimately reduces its antitumor efficiency.^54^

OA enhanced the apoptosis for CDDP/OA-LCC NPs against the HCC *via* Bad over expression by suppressing the AMPK/P13K/Akt/mTOR pathway activation^55^ as reported in our previous work shown in ESI Fig. S5.†^27^ AMPK pathway activation downgrades mTOR phosphorylation through dephosphorylating p70S6K (at Ser2448) and eukaryotic translation initiation factor 4E-binding protein 1 (4E-BP1) suppressing protein synthesis in cancer cells, and, by phosphorylating its upstream inhibitory regulator, TSC2 and Raptor; its regulatory subunit.

P53 pathway greatly regulates CDDP induced apoptosis, as its reduced activity lead to chemotherapy resistance. P53 pathway activation and stabilization results in enhanced Bax expression; a pro apoptotic protein involved with mitochondria in the intrinsic apoptotic pathway. P53; an important negative regulator of Bcl-2 which blocks the intrinsic apoptosis by preventing the pro-apoptotic proteins translocation from the cytosol to the mitochondria and cytochrome C release for apoptosis initiation *via* caspase activation.^56,57^ OA induced P53 stimulated caspase-mediated pro-apoptotic signaling pathway (shown in ESI Fig. S3†) which lead to the enhanced antitumor activity for CDDP/OA-LCC NPs against the HCC reported previously by our group.^27^ The basic mechanism underlying this apoptotic pathway is that OA induced an AMPK-dependent antitumor activity where AMPK activation results in increased p53 nuclear accumulation and apoptosis.^58^

Activated NF-κB is considered to be a major reason of CDDP resistance in many cancer cell lines by prompting a series of molecular reactions and up-regulating anti-apoptotic protein-encoding genes tempting cancer chemoresistance.^59,60^ Activated NF-κB also antagonizes p53 pathway functions resulting in chemotherapy resistance; probably through repressing p53 stabilization and activation, and transcriptional coactivators cross-competition. Hence, NF- κB pathway deactivation is of utmost importance in enhancing cellular sensitivity to CDDP in carcinoma cell lines.^15,61^ In our previous study, OA has been reported to deactivate NF-κB pathway;^27^ leading to the deactivation of its related anti-apoptotic proteins *i.e.* BCL-2 and X-linked inhibitor of apoptotic protein (XIAP) shown in ESI Fig. S5,† reversing the resistance of cancer cells for CDDP chemotherapy in our optimized formulation. The inhibitor of apoptosis proteins (IAPs), highly expressed in several cancers have been considered potential targets for cancer therapy. Down regulating IAPs expressions at both mRNA and protein levels in the tumors was found to reduce tumor growth. Several other groups have also reported that IAPs inhibition can reverse the resistance of cancer cells against chemotherapeutic drugs.^62^

These results and ours indicate that down regulating IAPs expressions in the tumors was found to reduce tumor growth, enhancing apoptosis for tumor-targeted therapy.

## 4. Conclusion

In summary, we have successfully developed a pH -responsive nanoplatforms based on CC NPs, which were then exploited as a nanocarriers for dual drugs loading (CDDP/OA) for an efficient combination chemotherapy against HCC. The pH-sensitive nanoparticles (CDDP/OA-LCC NPs) presented an excellent drug release profiles, remarkable site-specific tumor targeting and significant antitumor efficacy both *in vitro* and *in vivo*. Importantly, CDDP/OA-LCC NPs significantly reduced CDDP-induced damage to kidneys due to OA nephroprotective effects. Consequently, this pH-sensitive CC nanoparticulate system opens up a new possibility to develop combination chemotherapy strategies with limited side effects.

## Author contributions

Muhammad Waseem Khan: conceptualization, principal author, data curation, formal analysis, writing manuscript; Chenming Zou and Said Hassan: methodology, softwares applications; Fakhar Ud Din: performed the statistical analysis; Mahaman Yacoubou Abdoul Razak: contributed in the analysis of H & E staining assay; Asif Nawaz contributed in manuscript construction while Alam Zeb, Abdul Wahab and Sudhair Abbas helped in reviewing the final draft.

## Conflicts of interest

The author declares no competing financial interest.

## Abbreviations

CDDPOA-LCC NP: Lipid-coated cisplatin/oleanolic acid calcium carbonate nanoparticles
OA: Oleanolic acid
CDDP-CC: Cisplatin calcium carbonate cores
DOPA: 1,2-Dioleoyl-in-glycerol-3-phosphate
HSPC: Dehydrogenated soya phosphatidylcholine

## Supplementary Material

RA-012-D2RA00742H-s001

## References

[cit1] Xu G., Jin B., Xian X., Yang H., Zhao H., Du S., Makuuchi M., Pawlik T. M., Mao Y. (2021). Liver Cancer.

[cit2] Bray F., Ferlay J., Soerjomataram I., Siegel R. L., Torre L. A., Jemal A. (2018). Ca-Cancer J. Clin..

[cit3] Torbenson M. S. , Diagnostic Histopathology, 2021

[cit4] Han S., Lin F., Ruan Y., Zhao S., Yuan R., Ning J., Jiang K., Xie J., Li H., Li C. (2021). Int. Immunopharmacol..

[cit5] Jia Y.-Y., Zhang J.-J., Zhang Y.-X., Wang W., Li C., Zhou S.-Y., Zhang B.-L. (2020). Int. J. Pharm..

[cit6] Devarajan N., Manjunathan R., Ganesan S. K. (2021). Critical Reviews in Oncology/Hematology.

[cit7] Chraibi S., Rosière R., Larbanoix L., Gérard P., Hennia I., Laurent S., Vermeersch M., Amighi K., Wauthoz N. (2021). Eur. J. Pharm. Biopharm..

[cit8] Yu C., Dong H., Wang Q., Bai J., Li Y.-N., Zhao J.-J., Li J.-Z. (2021). Biomed. Pharmacother..

[cit9] Guan J., Tong X., Zhang Y., Xu F., Zhang Y., Liang X., Jin J., Jing H., Guo L., Ni X. (2021). Chem.-Biol. Interact..

[cit10] Lee D., Lee S. R., Kang K. S., Kim K. H. (2020). RSC Adv..

[cit11] Fang C.-y., Lou D.-y., Zhou L.-q., Wang J.-c., Yang B., He Q.-j., Wang J.-j., Weng Q.-j. (2021). Acta Pharmacol. Sin..

[cit12] Ma S., Xu H., Huang W., Gao Y., Zhou H., Li X., Zhang W. (2021). Frontiers in physiology.

[cit13] Tang Z.-Y., Li Y., Tang Y.-T., Ma X.-D., Tang Z.-Y. (2022). Biomed. Pharmacother..

[cit14] Woo J.-S., Yoo E.-S., Kim S.-H., Lee J.-H., Han S.-H., Jung S.-H., Jung G.-H., Jung J.-Y. (2021). Chem.-Biol. Interact..

[cit15] Chen F., Qin X., Xu G., Gou S., Jin X. (2017). Biochem. Pharmacol..

[cit16] Laraib U., Sargazi S., Rahdar A., Khatami M., Pandey S. (2022). Int. J. Biol. Macromol..

[cit17] Mendes B. B., Sousa D. P., Conniot J., Conde J. (2021). Trends Cancer.

[cit18] Sabu A., Lin J.-Y., Doong R.-A., Huang Y.-F., Chiu H.-C. (2021). Mater. Adv..

[cit19] Su X. , Zhang X., Liu W., Yang X., An N., Yang F., Sun J., Xing Y. and Shang H., 2021

[cit20] Tsakiris N., Fauvet F., Ruby S., Puisieux A., Paquot A., Muccioli G. G., Vigneron A. M., Préat V. (2020). J. Controlled Release.

[cit21] Chen Y., Chen C., Zhang X., He C., Zhao P., Li M., Fan T., Yan R., Lu Y., Lee R. J. (2020). Acta Pharm. Sin. B.

[cit22] Li Z., Huang J., Wu J. (2021). Biomater. Sci..

[cit23] Derakhshankhah H., Haghshenas B., Eskandani M., Jahanban-Esfahlan R., Abbasi-Maleki S., Jaymand M. (2022). Mater. Today Commun..

[cit24] Zheng B.-D., Huang Z.-L., Lv L.-L., Lan W.-L., Hu J.-Q., Li X., Zheng B.-Y., Ke M.-R., Huang J.-D. (2021). J. Mater. Chem. B.

[cit25] Zhu J., Jiao A., Li Q., Lv X., Wang X., Song X., Li B., Zhang Y., Dong X. (2022). Acta Biomater..

[cit26] Li H., Zhang X., Lin X., Zhuang S., Wu Y., Liu Z., Rong J., Zhao J. (2020). J. Mater. Chem. B.

[cit27] Khan M. W., Zhao P., Khan A., Raza F., Raza S. M., Sarfraz M., Chen Y., Li M., Yang T., Ma X. (2019). Int. J. Nanomed..

[cit28] Maleki Dizaj S., Barzegar-Jalali M., Zarrintan M. H., Adibkia K., Lotfipour F. (2015). Expert Opin. Drug Delivery.

[cit29] Kim S. K., Foote M. B., Huang L. (2013). Cancer Lett..

[cit30] Fanciullino R., Mollard S., Correard F., Giacometti S., Serdjebi C., Iliadis A., Ciccolini J. (2014). Pharm. Res..

[cit31] Naito S., Von Eschenbach A. C., Giavazzi R., Fidler I. J. (1986). Cancer Res..

[cit32] Khiati S., Luvino D., Oumzil K., Chauffert B., Camplo M., Barthélémy P. (2011). ACS Nano.

[cit33] Handa M., Beg S., Shukla R., Barkat M. A., Choudhry H., Singh K. K. (2021). J. Controlled Release.

[cit34] Gautam M., Santhiya D., Dey N. (2020). Mater. Today Commun..

[cit35] Gong M.-Q., Wu J.-L., Chen B., Zhuo R.-X., Cheng S.-X. (2015). Langmuir.

[cit36] Wang Z., Deng X., Ding J., Zhou W., Zheng X., Tang G. (2018). Int.
J. Pharm..

[cit37] Undevia S. D., Gomez-Abuin G., Ratain M. J. (2005). Nat. Rev. Cancer.

[cit38] Zheng N., Dai W., Du W., Zhang H., Lei L., Zhang H., Wang X., Wang J., Zhang X., Gao J. (2012). Mol. Pharmaceutics.

[cit39] Maeda H. (2015). Adv. Drug Delivery Rev..

[cit40] Guo P., Zhang N., Li J., Liu Y., Li Y., Wang X., Wang J., Wang Y., Wang A. (2022). Life Sci..

[cit41] Fukushima K., Futatsugi A., Maekawa M., Naito S., Okada A., Sugioka N. (2022). Biomed. Pharmacother..

[cit42] McSweeney K. R., Gadanec L. K., Qaradakhi T., Ali B. A., Zulli A., Apostolopoulos V. (2021). Cancers.

[cit43] El-Sayed R. M., El Gheit R. E. A., Badawi G. A. (2021). Life Sci..

[cit44] Wang S., Tang S., Chen X., Li X., Jiang S., Li H.-p., Jia P.-h., Song M.-j., Di P., Li W. (2020). Chem.-Biol. Interact..

[cit45] Volarevic V., Djokovic B., Jankovic M. G., Harrell C. R., Fellabaum C., Djonov V., Arsenijevic N. (2019). J. Biomed. Sci..

[cit46] Chen C., Ai Q., Wei Y. (2021). Int. Immunopharmacol..

[cit47] Atilano-Roque A., Wen X., Aleksunes L. M., Joy M. S. (2016). Toxicol. Rep..

[cit48] Liu J., Wang X., Liu R., Liu Y., Zhang T., Fu H., Hai C. (2014). Chem.-Biol. Interact..

[cit49] Liu C.-H., Huang X.-T., Li Y.-Y., Zheng X., Li N., Mi S.-Q., Wang N.-S. (2012). Zhongyaocai.

[cit50] Yuan L., Zhang L., Yao N., Wu L., Liu J., Liu F., Zhang H., Hu X., Xiong Y., Xia C. (2021). Phytomedicine.

[cit51] Potočnjak I., Šimić L., Vukelić I., Domitrović R. (2019). Food Chem. Toxicol..

[cit52] Yi J., Zhu J., Wu J., Thompson C. B., Jiang X. (2020). Proc. Natl. Acad. Sci..

[cit53] Liu R., Chen Y., Liu G., Li C., Song Y., Cao Z., Li W., Hu J., Lu C., Liu Y. (2020). Cell Death Dis..

[cit54] Fang S., Zhang P., Chen X., Liu F., Wang F. (2021). Front. Med..

[cit55] Nurcahyanti A. D. R., Jap A., Lady J., Prismawan D., Sharopov F., Daoud R., Wink M., Sobeh M. (2021). Biomed. Pharmacother..

[cit56] Wu X., Lu Y., Qin X. (2022). J. Ethnopharmacol..

[cit57] Bai H.-L., Kang C.-M., Sun Z.-Q., Li X.-H., Dai X.-Y., Huang R.-Y., Zhao J.-J., Bei Y.-R., Huang X.-Z., Lu Z.-F. (2020). Exp. Neurol..

[cit58] Fox M. M., Phoenix K. N., Kopsiaftis S. G., Claffey K. P. (2013). Genes Cancer.

[cit59] Kan Y., Liu J., Li F. (2020). Cancer Manage. Res..

[cit60] Li Y., Song X., Niu J., Ren M., Tang G., Sun Z., Kong F. (2021). Arch. Biochem. Biophys..

[cit61] Yu L., Li L., Medeiros L. J., Young K. H. (2017). Blood Rev..

[cit62] Hu F., Deng C., Zhou Y., Liu Y., Zhang T., Zhang P., Zhao Z., Miao H., Zheng W., Zhang W. (2022). Bioeng. Transl. Med..

