# Supplementary material for: Cisplatin and oleanolic acid Co-loaded pH-sensitive CaCO3 nanoparticles for synergistic chemotherapy
Source: RSC Adv. 2022 May 16;12(23):14808–18. doi: 10.1039/d2ra00742h (PMC9109477; doi:10.1039/d2ra00742h)
Supplement: RA-012-D2RA00742H-s001 [file RA-012-D2RA00742H-s001.pdf]

Electronic Supplementary Material (ESI) for RSC Advances.
This journal is © The Royal Society of Chemistry 2022

Cisplatin and Oleanolic acid Co-loaded pH-Sensitive $CaCO_3$ Nanoparticles for Synergistic Chemotherapy

Muhammad Waseem Khan[a,*], Chenming Zou[c], Said Hassan[c], Fakhar Ud Din[d], Mahaman Yacoubou Abdoul Razak[e], Asif Nawaz[f], Alam zeb[g], Abdul Wahab[h], Sudhair Abbas Bangash[i].

[a]Institute of Pharmaceutical Sciences, Khyber Medical University, Peshawar, Pakistan.
[b]School of Pharmacy, Tongji Medical College, Huazhong University of Science and Technology, Wuhan, Hubei 430030, China.
[c]Institute of Biotechnology and Microbiology, Bacha Khan University, Charsadda, Pakistan.
[d]Department of Pharmacy, Quaid-I-Azam University, Islamabad 45320, Pakistan.

[e]Department of Pathophysiology, School of Basic Medicine, Key Laboratory of Education Ministry of China for Neurological Disorders, Tongji Medical College, Huazhong University of Science and Technology, Wuhan 430030, China

[f]Faculty of Pharmacy, Gomal University, Dera Ismail Khan, Pakistan

[g]Riphah Institute of Pharmaceutical Sciences, Riphah International University, Islamabad, Pakistan.

[h]Department of Pharmacy, Kohat University of Science and Technology, Kohat, Pakistan.

[i]Faculty of Life Science, Department of Pharmacy, Sarhad University of Science and Information Technology, Peshawar, Pakistan.

*** Corresponding author.**

Postal addresses: Institute of Pharmaceutical Sciences, Khyber Medical University, Phase 5, Hayatabad, Peshawar, KP, Pakistan.
E-mail addresses: khanwaseem6065@gmail.com
Tel.: +92-3459146065

Supplementary Information

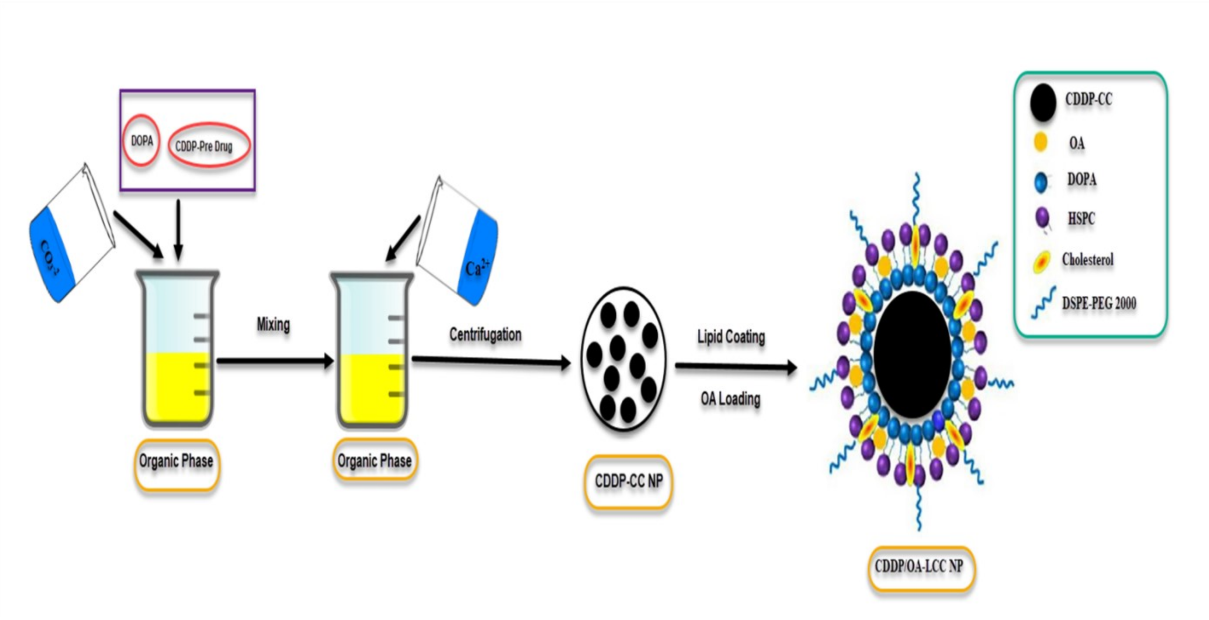

Supplementary Fig. S1. Schematic illustration of the formulation of lipid coated cisplatin/oleanolic acid co-loaded calcium carbonate nanoparticles (CDDP/OA-LCC NPs).

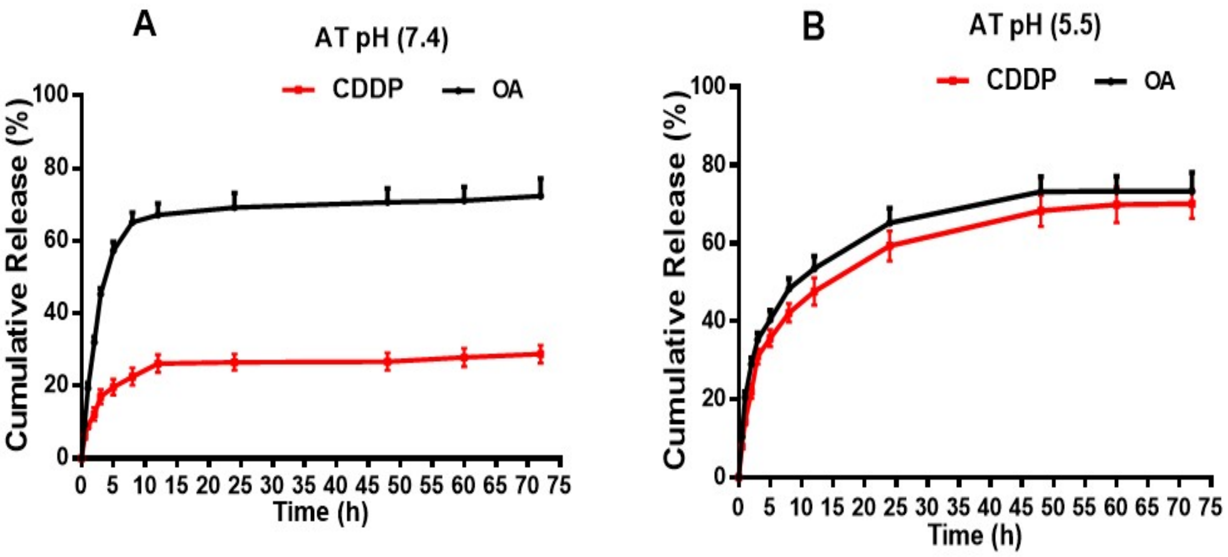

Supplementary Fig. S2. In vitro drug release profiles of CDDP and OA from the CDDP/OA-LCC NPs in PBS (72 h). (A) At pH 5.5; (B) At pH 7.4.

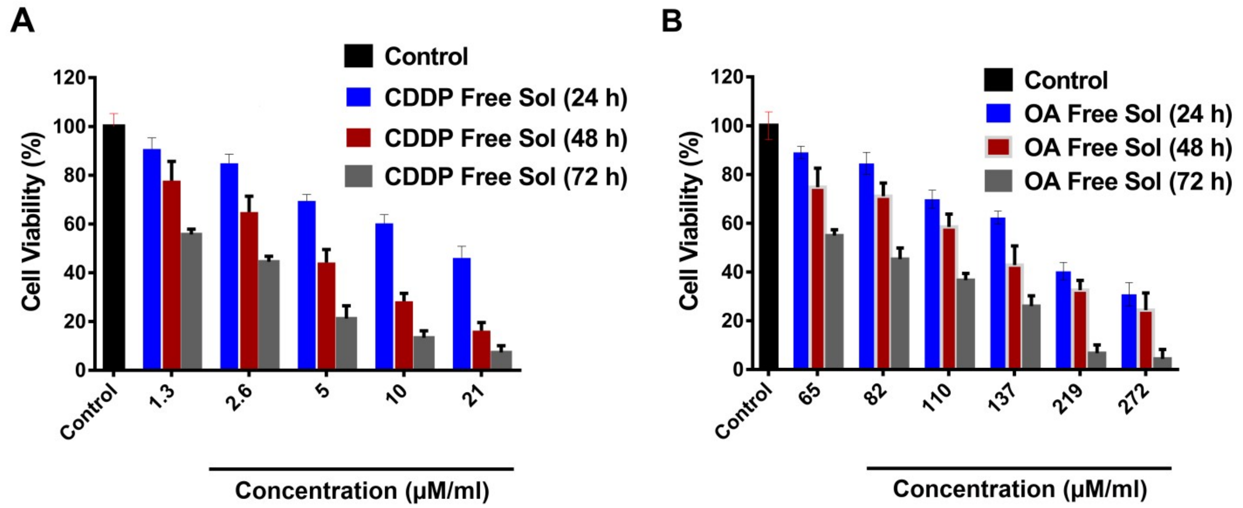

Supplementary Fig. S3. Cytotoxicity assay of free CDDP and free OA against HepG2 cells.

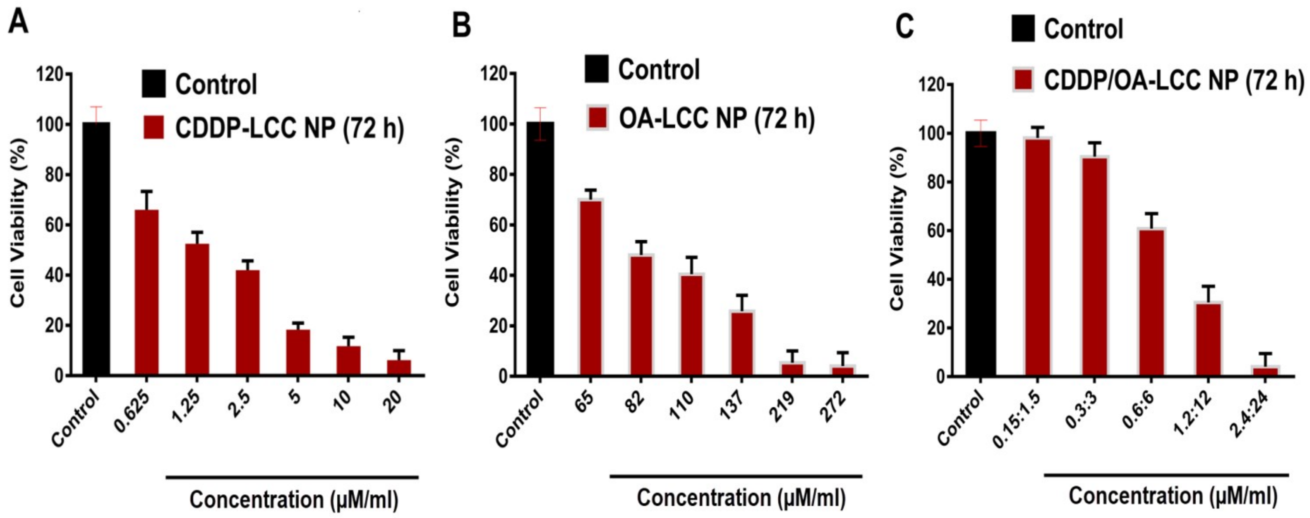

Supplemenatry Fig. S4. Cytotoxicity assay of the nanoparticles against HepG2 cells (72 h). (A) CDDP-LCC NPs MTT assay; (B) OA-LCC NPs MTT assay; (C) CDDP/OA-LCC NPs MTT assay with fixed ratios. Data presented as mean ± SD, n=5.

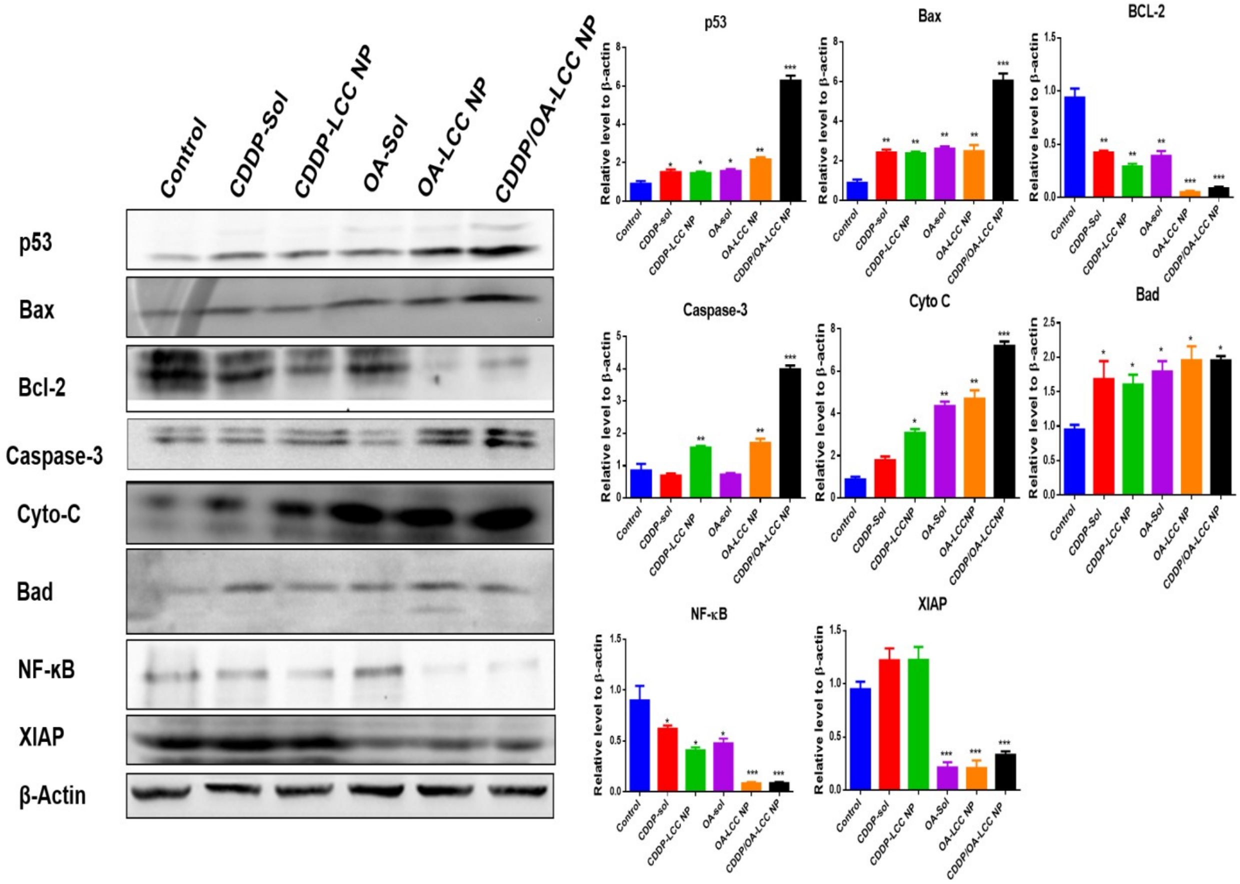

Supplementary Fig. S5. Western blot analysis of protein levels (p53, Bax, Bad, Cyto-C, caspase-3, NF-κB, Bcl-2 and XIAP) after treating HepG2 cells with CDDP-Sol, CDDP-LCC NP, OA-Sol, OA-LCC NP, CDDP/OA-LCC NP *in vitro*. β-actin was used as a loading control. Quantification of protein level using Image J. Data presented as mean ± S.D. (n=3).
